# Image-Based Modeling Reveals Dynamic Redistribution of DNA Damage into Nuclear Sub-Domains

**DOI:** 10.1371/journal.pcbi.0030155

**Published:** 2007-08-03

**Authors:** Sylvain V Costes, Artem Ponomarev, James L Chen, David Nguyen, Francis A Cucinotta, Mary Helen Barcellos-Hoff

**Affiliations:** 1 Life Sciences Division, Lawrence Berkeley National Laboratory, Berkeley, California, United States of America; 2 Johnson Space Center, National Aeronautics and Space Administration, Houston, Texas, United States of America; 3 Universities Space Research Association, Houston, Texas, United States of America; University of California, United States of America

## Abstract

Several proteins involved in the response to DNA double strand breaks (DSB) form microscopically visible nuclear domains, or foci, after exposure to ionizing radiation. Radiation-induced foci (RIF) are believed to be located where DNA damage occurs. To test this assumption, we analyzed the spatial distribution of 53BP1, phosphorylated ATM, and γH2AX RIF in cells irradiated with high linear energy transfer (LET) radiation and low LET. Since energy is randomly deposited along high-LET particle paths, RIF along these paths should also be randomly distributed. The probability to induce DSB can be derived from DNA fragment data measured experimentally by pulsed-field gel electrophoresis. We used this probability in Monte Carlo simulations to predict DSB locations in synthetic nuclei geometrically described by a complete set of human chromosomes, taking into account microscope optics from real experiments. As expected, simulations produced DNA-weighted random (Poisson) distributions. In contrast, the distributions of RIF obtained as early as 5 min after exposure to high LET (1 GeV/amu Fe) were non-random. This deviation from the expected DNA-weighted random pattern can be further characterized by “relative DNA image measurements.” This novel imaging approach shows that RIF were located preferentially at the interface between high and low DNA density regions, and were more frequent than predicted in regions with lower DNA density. The same preferential nuclear location was also measured for RIF induced by 1 Gy of low-LET radiation. This deviation from random behavior was evident only 5 min after irradiation for phosphorylated ATM RIF, while γH2AX and 53BP1 RIF showed pronounced deviations up to 30 min after exposure. These data suggest that DNA damage–induced foci are restricted to certain regions of the nucleus of human epithelial cells. It is possible that DNA lesions are collected in these nuclear sub-domains for more efficient repair.

## Author Summary

DNA damages are daily cellular events. If such events are left unchecked in an organism, they can lead to DNA mutations and possibly cancer over a long period of time. Consequently, cells have very efficient DNA repair machinery. Many studies have focused on the different molecular factors involved in the repair machinery, neglecting to consider the spatial context where damage occurs. Therefore, little is known about the role the nuclear architecture might have in the DNA damage response. In this study, we introduce computer modeling and image analysis tools in order to relate the position of DNA damage markers to morphologically distinct regions of the nucleus. Using these tools, we show that radiation-induced damages locate preferentially in non-condensed DNA regions or at the boundary of regions with condensed DNA. These results contradict the current dogma that the molecular response to randomly generated DNA damages is independent of their nuclear locations. Instead, this suggests the existence of repair centers in the nucleus. Overall, our approach shows that nuclear architecture plays a role in the DNA damage response, reminding us that the nucleus is not simply a soup of DNA and proteins.

## Introduction

DNA damage induced by ionizing radiation (IR) elicits microscopically visible nuclear domains (i.e., foci) marked by recruitment of certain proteins (e.g., 53BP1) or by particular modifications such as histone phosphorylation (e.g., γH2AX) or as a result of both (e.g., phosphorylated ATM, ATMp) [1–10]. Radiation-induced foci (RIF) are believed to form at or adjacent to sites of DNA damage. However, the use of RIF as an unequivocal indicator of double strand break (DSB) is problematic. The readout of RIF is complex as it is based on optical limitations during image acquisition (e.g., point-spread function (PSF)), non-homogeneity of the detector (i.e., nucleus), and biological kinetics. Our previous work and that of others have suggested that the detection of RIF reflects several factors: (1) the severity of the damage, (2) the efficiency of damage recognition, (3) repair capacity, and (4) the biological function of the specific RIF proteins [7,11–14]. Furthermore, some reports suggest that there are nuclear regions that are excluded from forming RIF. More specifically, in studies using densely ionizing particles that would lead to continuous DSB along their trajectories, nuclei showed discontinuous MRE11 RIF, with large gaps (>1 μm) in regions where DNA was present [15]. Finally, others have shown that some types of RIF are not necessarily associated with DSB [12].

In studying DNA damage responses using RIF, how can one interpret results if RIF are not necessarily related to DSB? To sort out these discrepancies, one could compare the spatial distributions of RIF from different radiation qualities and relate them to the expected energy deposition described by physical attributes. We propose to compare γ-rays and high energy particles (HZE), which lead to very distinct spatial distributions of energy deposition. HZE are high-LET radiation and deposit their energy in random clusters along a linear path [16,17]. Their complex physical interactions with cells have been well characterized and therefore can be modeled [18]. Cells exposed to HZE provide an excellent model in which to study the relationship between chromatin patterns and energy deposition since energy deposition, and therefore image analysis, is reduced to essentially 1-D linear profiles in a plane of the nucleus. In contrast, γ-rays are low-LET radiation that deposit energy uniformly in a small volume and thus induce single DSB randomly across the nucleus. While these events are easily modeled, characterizing low-LET RIF spatially is more complex since it requires 3-D image analysis of nuclei.

We have previously used parameters determined by fitting data from DNA fragments sizes measured in pulsed-field gel electrophoresis (PFGE) experiments to show that radiation-induced DSB are generated as a stochastic process [19–21]. This assumption has led to computation models that simulate the production of DSB in hypothetical spatial geometries [22,23]. In this study, we further refine these models to include higher order nuclear territories such as euchromatin and heterochromatin. Artificial microscope images of RIF and nuclei can then be generated by including optical limitations of light microscopy. We then show that we can predict the DNA damage pattern for any given radiation by generalizing the theoretical model to an image-based model. The central point is to use the image-based model to test the controversial equivalence between RIF and DSB. If RIF are in fact DSB observed at a much lower resolution, then we would predict similar spatial distribution and frequencies. However, our results show that within 5 to 30 min following exposure to high-LET and low-LET radiation, RIF distributions deviate from the predicted DSB distribution. We further show that RIF non-randomly locate in specific regions of the nucleus. This suggests that nuclear organization modulates the DNA damage response of human epithelial cells, which has important implications for understanding DNA damage response and repair mechanisms.

## Results

### Generation of Pseudo RIF in Synthetic Nuclear Images

**Figure 1:**
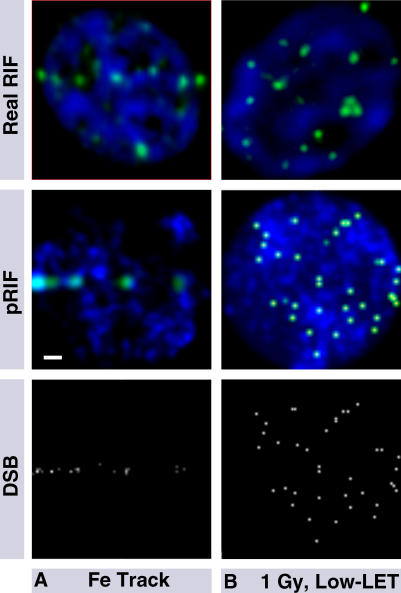
Comparison between Simulated and Experimental Images for Different Types of Radiation (A) Shows RIF within a nucleus traversed by high energy Fe ions (1 GeV/amu Fe). (B) shows a nucleus exposed to photons. Shown at the top are RIF images of nuclei taken from a microscope (DAPI in blue and DNA damage marker γH2AX in green) 5–10 min post-IR. Middle images are pRIF microscopic images at the same magnification. These pRIF images are generated by blurring DNA damage simulations for equivalent doses of radiation with the PSF of the optic used. For pRIFs, the blue channel shows the resulting nuclear density and the green channel shows DSB. If the PSF is omitted from the simulation, DSB locations can be better resolved, as shown in the gray images at the bottom of each panel (brightness proportional to the number of breaks within each pixel). Scaling bar shown in white is 1 μm wide.

In this approach, the 3-D space was divided into cubic pixels of the size equal to that of microscopy image (i.e., 0.16 μm pixels). DNA in the simulated nucleus was arranged into two types of intermittent bands: dense regions of DNA based on random-walk geometry (heterochromatin), and low-density homogenous regions (euchromatin). DNA double strand breaks (DSB) were simulated by Monte Carlo simulations for single traversal of 1 GeV/amu Fe ions or for exposure to 1 Gy of low-LET radiation [24]. Theoretical DSB are absolute, whereas the visualization and quantitation of RIF are subject to optical limitations during image acquisition. Therefore, to more closely approximate RIF from theoretical DSB, DSB locations were blurred by applying a Gaussian filter with σ = 0.16 μm, determined by the PSF of the microscope. The resulting images were similar to images collected experimentally as illustrated in Figure 1. Applying the Gaussian blurring (Gaussian convolution) to the DSB frequency image produces images with foci-like objects (Figure 1), which we refer to as pseudo-foci (pRIF). pRIF reflects the appearance of DSB at light microscope resolution should they bind enough antibody to emit sufficient fluorescence to be detected. Both high-LET (1 GeV/amu Fe ion tracks) and low-LET simulated images are depicted and compared with real images labeled for the DNA damage marker γH2AX (Figure 1A and 1B, respectively). One can appreciate from Figure 1A the fact that many close-by DSB along high-LET tracks cannot be resolved and end up appearing as large foci, a phenomenon that has been reported previously on experimental data [7,11].

### Comparing pRIF Frequencies and Experimental RIF Frequencies

To validate whether the frequency of theoretical pRIF occurring in synthetic nuclei are comparable to actual measurements, the frequency of RIF was measured for different DNA damage markers (53BP1, γH2AX, and ATMp), and within the first hour following exposure to 1 Gy of either low-LET or high-LET radiation. The measured frequencies are shown in Table 1. For high-LET radiation, we observed excellent agreement between pseudo- and measured RIF, leading to a maximum of 0.73 RIF/μm 4.5 min following exposure to 1 GeV/amu Fe. On the other hand, consistent with our previous findings and those of others [7,11–15], the maximum measured frequencies for low-LET RIF in 3-D volume occurred 30–60 min after exposure and was 60% lower than predictions. Unfortunately, comparing pRIF to RIF at such late time points is difficult to interpret as DSB repair is significant over the first hour following irradiation. Comparing at earlier time points is not ideal either, as RIF frequencies at 4.5 min post-IR were even lower (70% lower than prediction, unpublished data). Note that the PFGE data used to predict DSB in our model do not distinguish DSB from heat-labile sites [25]. However, removing DSB from these sites would not be sufficient to have predictions that match measurements for low LET.

**Table 1:**
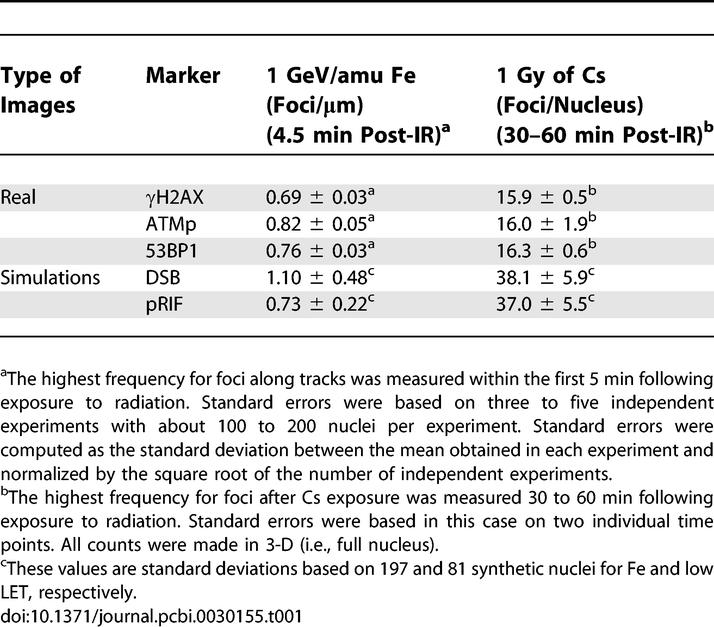
RIF Frequencies in Experimental Data and Simulations for High-LET and Low-LET Radiation

Another point illustrated by these simulations is the effect of the optical PSF (see Materials and Methods) on visualizing DSB by light microscopy. For low-LET simulations, there is little variance between predicted DSB and pRIF. In this case, a 1:1 correspondence between DSB and pRIF is expected since such sparse damage events remain separate after optical blurring. On the other hand, for HZE, the frequency of pRIF and theoretical DSB, both generated in synthetic nuclear images, differ by 30%. The 30% loss between DSB and pRIF is due to clustered DSBs that are not resolvable by light microscopy. Since RIF frequencies match pRIF frequencies for HZE, experimental RIF in this case must represent clustered DSBs at a resolution lower than the original scale of DNA breaks (nm versus μm for microscopy). Interestingly, this also suggests that if damages are more complex or span over a larger DNA range (i.e., cluster of DSBs), it will rapidly induce RIF (within 5 min), whereas a single DSB may not always lead to RIF and/or may have a slower formation kinetic.

### Use of an Imaging Approach To Predict DSB Location in Real Images

pRIF predictions presented previously are based on DNA patterns from hypothetical nuclei, modeled at the nm scale. As described in methods, DSB are predicted to be dependent on DNA density. As such, any given nucleus has a unique DNA imaging pattern and a unique set of spatial probability for radiation-induced DSB. Therefore, we cannot directly predict the DSB patterns in real images and compare their spatial distributions to RIF using a theoretical model of the nucleus.

To answer such need, we introduce here an imaging methodology that can predict DSB location for any given DNA nuclear pattern from real images. This methodology is based on the same Monte Carlo concepts described in Materials and Methods for the generation of DSBs in artificial nuclei: i.e., the probability of a DSB at a given location is proportional to the DNA density at the same location. This statement is true at high resolution, if each pixel could contain a single DSB. However, this rule may not be true at the sub-micron resolution of a microscope, where each pixel encompasses large amounts of DNA, where neighboring pixels are slightly correlated [26], and where individual DSBs are not always resolvable.

**Figure 2:**
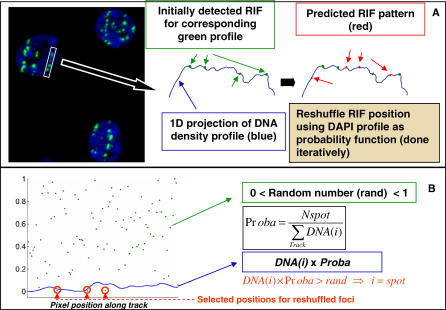
Illustration of Image Manipulation to Predict the Average DNA Damage Pattern along a Track for a Given DNA Density Profile (A) Shows a typical image of cells that have been traversed with 1 Gy of 1 GeV/amu Fe ions. After manually selecting a region that contains a clear track, foci identification and reshuffling is done as depicted by the cartoon. Foci detection is done automatically via in-house image algorithm (see Materials and Methods). (B) Further illustrates the mathematical approach used (i.e., Monte Carlo concept), where the probability of damage at a pixel location is proportional to the DNA density at the same location. This process is done iteratively (i.e., 50 randomizations per nucleus analyzed) to give a reasonable average break distribution. For each iteration, RIF position is determined by a probability less than that determined by DNA density (blue line).

We illustrate our imaging approach using high-LET radiation data, where RIF tracks demarcate the damaged nuclear regions. Within these tracks, spatial 1-D profiles of DNA density and the number of foci (*Nspot*) can be determined (see Figure 2A). Assuming that the probability of a RIF at a given location along the track is proportional to the DNA density at that location, one can then compute the probability per unit pixel intensity to have a focus as follows:

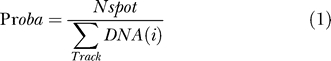
where *DNA*(*i*) is the DNA density at position *i* along the indexed pixel of the track. The probability *P_DSB_* of a DSB at any given pixel location along the track is then:

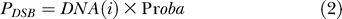



If this probability is greater than a random value taken between 0 and 1, then a focus is generated at this location. Applying this approach to all the pixels along the track, we can generate a set of new foci referred to as “reshuffled foci” (see Figure 2B).

**Figure 3:**
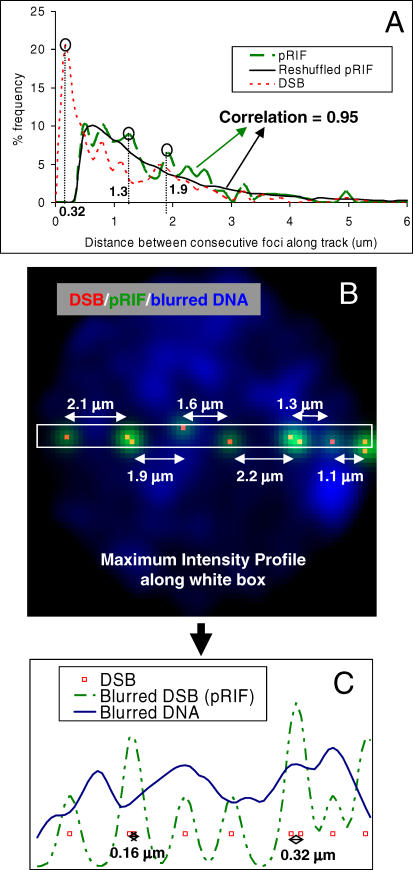
Comparison of Image Prediction and Monte Carlo Simulation for DNA Damage Distance Distribution (A) shows the distribution of distance between consecutive foci along the track for a set of 197 simulated nuclei exposed to a theoretical 1 GeV/amu Fe track. (B) illustrates a simulated nucleus: DSB are shown in red, DNA densities and DSB blurred with the PSF in blue and green, respectively. pRIF, blurred DSB, are identified by detecting maxima along the intensity profile (C) sampled over a narrow strip of the image in (B). These profiles are obtained by computing the maximum intensity projection of a 0.8-μm thick line aligned with the particle track. Some of the distances reported for this illustrated track are also shown in (B) and (C) and labeled correspondingly in (A). The average DSB distance distribution over all 197 nuclei is shown by the red dotted curve showing an expected Poisson-like distribution. The corresponding distance distribution for pRIF is shown as the dashed green curve and is similar to the DSB distribution except for the frequency of close-by foci that have diminished (i.e., need at least more than two-pixel gap to be separate, which corresponds to 0.48 μm). We could reproduce this behavior (dark solid curve) by simply randomizing pRIF along the track using the DNA profile as a probability for DNA damage (as described in Figure 2).

The validity of this image methodology was tested on the set of Fe track simulations described previously in Table 1. The damage pattern along the artificial tracks was characterized by measuring distances between consecutive pRIF (Figure 3B and 3C). Reshuffling pRIF position led to spatial distributions similar to the original pRIF (Figure 3A) and thus confirmed that this image manipulation is an accurate way to predict damage distribution in a microscope image. On the other hand, even though the main shape of the pRIF distribution was conserved in the reshuffled pRIF distribution, the smaller variations were lost. These irregularities in the distribution probably reflect the lack of uniformity of DNA density at a much higher resolution since the same irregularities were also apparent in the DSB distribution predicted by modeling.

To summarize, the probability of generating DSB remains proportional to DNA density at a lower resolution (i.e., sub-micron), and therefore DNA density measured by light microscopy along a track can be used as a DNA damage probability.

### Reshuffling Experimental RIF To Predict DNA Damage Pattern for a Given Nucleus

As we validated in silico our imaging approach to predict DNA damage patterns along the HZE track, we next compared actual RIF with predicted RIF in situ for the same radiation quality. The nuclear dye 4′,6-diamidino-2-phenylindole (DAPI) binds to AT base pairs or to single strands by electrostatic interaction. Therefore, one can assume as a first-order approximation that the pixel intensity in a DAPI image is proportional to the DNA concentration at that location. We therefore used DAPI as an indicator of DNA densities in real images. Primary antibodies to ATMp, γH2AX, or 53BP1 and fluorescently labeled secondary antibodies were used to detect RIF.

**Figure 4:**
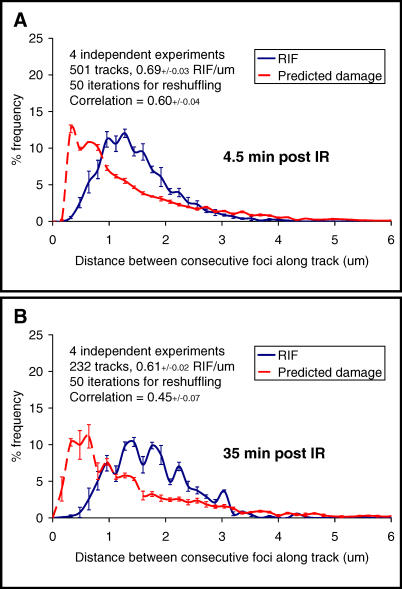
Comparing Theoretical and Experimental DNA Damage Pattern along 1 GeV/amu Fe Track Average distributions of distances between consecutive foci along Fe tracks are plotted at 4.5 min and 35 min following 1 Gy exposure (blue solid lines, (A) and (B), respectively). Error bars are standard errors based on four independent experiments. For each individual track analyzed from real data, foci were counted and their positions were then randomized based on DNA profiles, described previously, to generate a theoretical distribution pattern (red dashed lines). Measuring the correlation between theoretical and experimental distributions, we observe a decrease of correlation between these two time points, from 0.6 to 0.45. These data indicate that as early as 4.5 min following exposure to radiation, foci positions deviate from a theoretical random behavior by 40% and this tendency increases over the next 30 min with a 55% loss of correlation.

**Figure 5:**
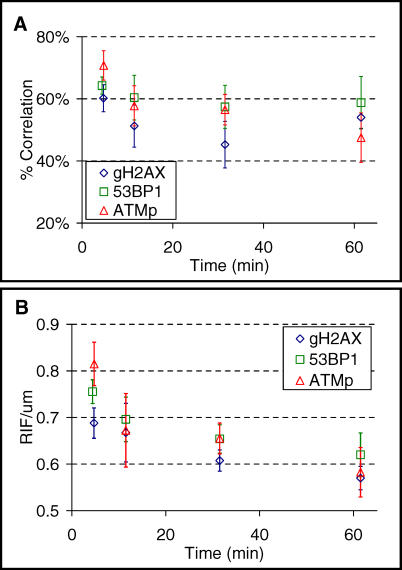
Spatial Foci Pattern Increasingly Deviates from Prediction Following 1 Gy of 1 GeV/amu Fe Exposure (A) Plots the correlation between theoretical and experimental distributions of distances between consecutive foci along Fe tracks over the first hour following exposure to radiation. All DNA damage markers used (i.e., γH2AX, ATMp, 53BP1) show the same loss of correlation to randomness over time. (B) Shows the corresponding foci frequencies, depicting a rapidly decreasing curve indicative of DNA repair.

Our results show that distribution of foci along tracks in cells irradiated by 1 GeV/amu Fe deviate from truly random distribution. This deviation increases with time independently of the marker used for damage. This is illustrated in Figure 4 for γH2AX, where the distance distribution between consecutive foci is compared with the distribution of reshuffled γH2AX foci at 4.5 and 30 min following exposure to 1 GeV/amu Fe. As early as 4.5 min post-IR, only 60% of the RIF distribution correlated with the distribution of predicted damages (i.e., reshuffled RIF). We predicted that the majority of damages would be less than 1 μm apart. Instead, the majority of measured RIF were more than 1 μm apart. The exclusion of close-by foci in the experimental data also increased with time. Figure 5A summarizes these results for all the time points (4.5, 11.5, 31.5, 61.5 min) by plotting the average correlations measured between predicted (i.e., reshuffled foci) and measured distance distribution for the different DNA damage RIF. All RIF show the same trend with an increasing deviation from random distribution over the first hour following exposure to 1 Gy of 1 GeV/amu Fe. Note that the predicted RIF distance distribution is based on reshuffling the exact same number of detected RIF for each individually analyzed track. Therefore, the loss of close-by foci cannot be attributed to their diminishing frequency (shown in Figure 5B). These results show in fact an increase in organization suggesting DNA damage clustering into self-excluding sub-regions of the nucleus within an hour following exposure to HZE, as suggested by others [27].

### Relative DNA Image Measurements

**Figure 6:**
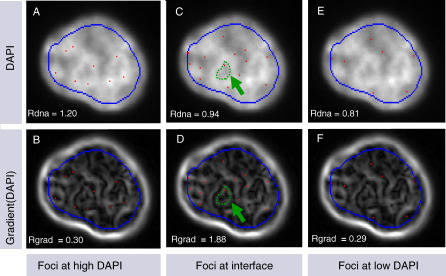
Illustration of *Rdna* and *Rgrad* Measurements Three hypothetical foci patterns over the same nucleus are illustrated with their corresponding *Rdna* and *Rgrad* values. Upper images (A,C,E) are overlays of the DAPI image with the center of hypothetical foci (in red). Lower images (B,D,F) are overlays of the foci location with the gradient image of DAPI. The gradient operator is often used in imaging as an edge detector. To illustrate this, the green arrow in (C) delineates the contour of the edge of a bright DAPI region. One can see in the corresponding gradient image in (D) that the same contour correlates to a bright gradient region. *Rdna* measures the ratio of the mean nuclear intensity at the foci locations over the mean intensity of the full nucleus. *Rgrad* measures the same ratio on the gradient image. Because the boundary of the nuclear image creates a strong gradient intensity, a conservative contour is used for nuclear segmentation (shown in blue) to avoid an edge effect when calculating *Rdna* and *Rgrad*. In (A) and (B), foci are placed in areas of surrounding high nuclear density. The surrounding high density keeps the foci distal from areas of density change, thus we see the foci lie in low-intensity regions in the corresponding gradient image. This results in *Rdna* above 1 and *Rgrad* below 1. By manually placing foci at different locations with respect to DNA density regions, we show that *Rdna* is high when foci are located in bright regions of the nucleus (A) and (B); *Rgrad* is high when foci are located at the interface of bright and dim regions of the nucleus (C) and (D); and *Rdna* is low when foci are located in dim regions of the nucleus (E) and (F).

Given the deviation of RIF from random distribution over time, we can then ask if foci relocate in regions of the nucleus with specific morphological features. To do so, we introduce a set of imaging parameters that ascertains the position of foci with respect to DNA density. This set of parameters can also be measured in any spatial dimension (i.e., line profiles, surfaces, or volumes). Figure 6 illustrates the approach on a given nucleus (i.e., center slice of a nucleus—DAPI stain). Using automatic spot detection (see Materials and Methods), we consider the center of RIF as the brightest pixel in its vicinity. One can then compute the mean DNA density signal at the centers of all RIF in one nucleus and normalize it to the mean nuclear DNA density to get a relative DNA density value at these locations. We thus define the relative density of DNA at the foci locations as follows:

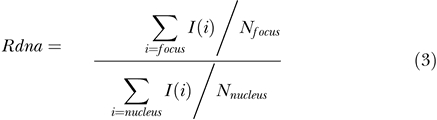
where, *I*(*i*) is the intensity at pixel location *i*, *N_focus_* is the number of foci, and *N_nucleus_* is the number of pixels in the nucleus (note, *I = focus* refers to the brightest pixel in an identified focus).

The DNA gradient is a good indicator of edges between high and low DNA density regions. Therefore, we can also compute the relative position of foci with respect to the surface of dense DNA regions by evaluating the DNA gradient value where foci are detected. This leads to the parameter *Rgrad* as described in Figure 6. Similarly to *Rdna* definition, *Rgrad* is the mean DNA gradient at the foci location normalized to the mean gradient over the full nucleus, defined as follows:

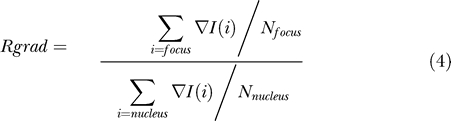
where ∇*I*(*i*) is the Euclidian norm of the gradient vector of *I*(*i*) at pixel location *i*. Note that the mask used to compute *Rdna* and *Rgrad* is based on a conservative segmentation of the nucleus. The contour of the nucleus is defined as a region well inside the nucleus (i.e., 0.48 μm inward of the nuclear boundary). This conservative segmentation is necessary to remove edge effect when computing the gradient of the nuclear DAPI image.

If foci preferentially locate in bright regions of the DNA, *Rdna* will be greater than 1. Similarly, if foci locate preferentially at the interface between bright and dim regions of the DNA, *Rgrad* will be greater than 1. We can further test these concepts by measuring *Rdna* and *Rgrad* in the simulated data previously discussed where we know what to expect. For 1 GeV/amu Fe simulations, we logically find that *Rdna* values for pRIF are greater than one (see Table 2), reflecting the fact that generation of damage is proportional to the amount of DNA: i.e., the more DNA, the more likely radiation will produce a break. On the other hand, *Rgrad* values are also larger than 1. This result at first hand might look surprising as one would assume that there should be no preferential location of DSB with respect to nuclear interfaces. However, this result simply reflects the fact that gradient values are larger in denser regions of DNA. We can also compare the relative measurements between observed foci and reshuffled foci to verify that our image-based prediction of DNA damage leads to the same values. The ratio of *Rdna* (*R*) and *Rgrad* (*Rg*) between simulated pRIF (indexed 1) and reshuffled pRIF (indexed 2) are shown in Table 2 (i.e., *R1/R2* and *Rg1/Rg2*). These ratios are very close to 1, indicating a foci pattern for pRIF that matches the expected random distribution of DNA damage.

**Table 2:**
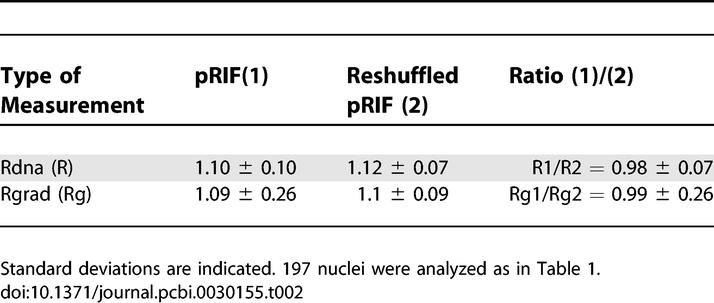
Reshuffling Simulated Foci Positions along Synthetic Tracks Lead to the Same Relative DNA Values as Simulations

DNA damage induced by low-LET radiations are scattered throughout the nucleus. Therefore, in order to predict a DSB imaging pattern for low LET, we need to generalize the reshuffling approach in 3-D. This can be done in the same manner it was done for high-LET tracks using DNA density at any pixel in the nucleus as a probability to have damage at that location. Similarly to HZE, the validity of this approach was tested by comparing the *Rdna* and *Rgrad* values for pRIF and reshuffled pRIF in the low-LET simulations from Table 1. Reshuffling pRIF over the full nucleus for low-LET data also led to similar *Rdna* and *Rgrad* values than for pRIF (i.e., *R1/R2* of 1.05 ± 0.09 and *Rg1/Rg2* of 0.96 ± 0.11). Therefore, reshuffling foci in 3-D remains a valid approach to predict DSB patterns for low LET.

### Application of Relative DNA Measurements to Experimental Data

**Figure 7:**
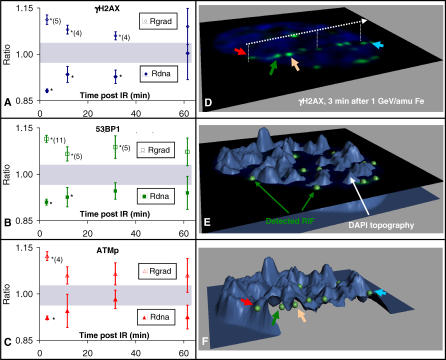
*Rdna* and *Rgrad* Computation Confirm Rapid Relocation to Dim–Bright Nuclear Interfaces with a Lower Proportion of Foci in the High DNA Density Regions after Exposure to 1 Gy of 1 GeV/amu Fe Measured *Rdna* and *Rgrad* normalized to predicted values are graphed in (A–C). For all DNA damage markers analyzed here, all *Rgrad* ratios are above 1 and Rdna ratios are below one. This indicates a tendency of RIF to locate themselves at the interface between high and low DNA density regions and preferably in the low DNA density regions. This tendency is stronger within the first 10 min following exposure to radiation and statistically significant for γH2AX and 53BP1 for the first 30 min post-IR (significance is labeled by an asterisk with the number of independent experiments in parentheses, statistical test based on *t-*test between measured averages and predicted ones. Predictions are based on reshuffling original RIF. Based on that test, a 95% confidence interval for expected normalized ratios is shown as the gray area). For ATMp, only the earliest time point was statistically significant, indicating a return to normality much faster than the other markers. A representative nucleus 3 min post-IR is shown in (D), with γH2AX RIF appearing as a green signal and DAPI shown as blue. The white dashed arrow indicates the traversal of one Fe particle, and small solid-color arrows indicate specific RIF. The same nucleus is seen in (E) with the DAPI intensity displayed in a 3-D topographic blue surface and segmented γH2AX RIF shown as green beads. (Rendering done with Bitplane, http://www.bitplane.com/). (F) shows the same topographic view, sectioned along the particle trajectory to better appreciate the position of RIF with respect to the DAPI intensity profile. For orientation purposes, the same RIF shown with solid color arrows in (D) are shown in (E) and clearly illustrate the preferred location of RIF at the interface between high and low DNA density regions.

The kinetics of normalized *Rdna* and *Rgrad* values for cells exposed to 1 GeV/amu Fe are shown in Figure 7A–7C. *Rdna* and *Rgrad* ratios along tracks confirm our previous finding that γH2AX and 53BP1 RIF spatial distributions deviate from the predicted nuclear locations of DNA damage. *Rdna* averages were less than the predicted *Rdna* (ratio less than 1) at all time points, as early as 4.5 min post-IR. Similarly, the *Rgrad* averages were always greater than predicted. Thus, on average, RIF were located in lower DNA density regions than where DSBs were expected to occur, and RIF tended to be located at the interface between high and low DNA density regions (see Figure 7D–7F for illustration of phenomenon). Interestingly, ATMp foci showed a slightly different dynamic. Although ATMp RIF localized to chromatin regions of slightly less DNA at the interface of high and low densities at the earliest time point, by 10 min post-IR the measured *Rdna* and *Rgrad* were 1, i.e., similar to that predicted for DSB.

**Figure 8:**
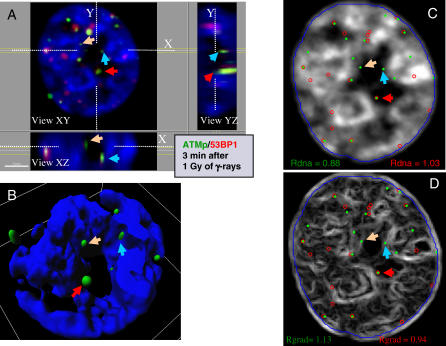
Representative RIF Distribution Following Low-LET Radiation Exposure DAPI is shown in blue, ATMp in green, and 53BP1 in red. (A) shows a representative 3-D image of a nucleus in orthogonal cross-section views. The same nucleus is seen in (B) as a 3-D surface rendering with only DAPI and ATMp RIF being segmented (i.e., blue and green surfaces, respectively; rendering done with Bitplane). This nucleus clearly shows the preferred location of ATMp RIFs at the interface between high and low DNA density regions. Different ATMp RIFs located at these interfaces are shown by colored arrows. (An arrow of the same color represents the same focus.) (C) and (D) overlay the identified locations of ATMp and 53BP1 RIFs (green disk and red circles, respectively) with the DAPI intensity and gradient images, respectively. *Rdna* and *Rgrad* values are given for each panel with colored text corresponding to the protein. One can see in (D) that the green RIFs shown by arrows are along high-gradient contours, which are reflected by a high *Rgrad* value. In contrast, the red RIF seem to locate themselves fairly randomly over the full nucleus, as reflected with *Rdna* and *Rgrad* values close to 1.

Analysis of low-LET data required specific imaging considerations as discussed previously for the 3-D generalization of our approach. In addition, due to the poor resolution of real conventional microscope images in the Z direction, *Rdna* and *Rgrad* computations were done only on the best focal plane of 3-D image stacks for each cell (see Figure 8). Our results showed that *Rdna* values were all slightly lower than the predicted values for all three RIF as summarized in Table 3. Even though deviations from prediction were small, the robustness of these measurements was evident in the very small standard errors (i.e., 0.3%–2%), allowing the detection of very subtle differences. Such small errors led to statistical significance for γH2AX *Rdna* and *Rgrad* values early after exposure to radiation. All time points were also significant for the *Rgrad* values of 53BP1.

**Table 3:**
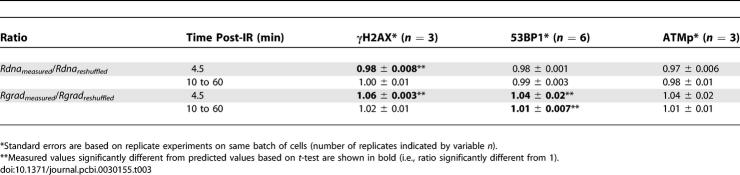
Experimental Relative DNA Measurements Normalized to Predicted-for Exposure for 1 Gy of γ-Rays

### Increased Co-Localization between RIF during the First Ten Minutes Following Exposure to Radiation

**Figure 9:**
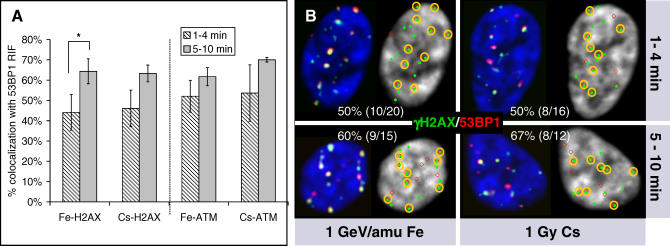
Increased Co-Localization of DNA Damage Markers Rapidly after Exposure to Radiation (A) is an analysis summary of γH2AX and ATMp co-localization with 53BP1 done in 3-D. Two RIFs are considered co-localized if the distance between their centers is less than or equal to 0.48 μm. As described in Materials and Methods, RIF centers are determined as the brightest pixel within each spot. The γH2AX co-localization is illustrated in (B) with representative images of different time points and different type of radiations. γH2AX and 53BP1 foci locations are shown as green disks and red circles, respectively. Co-localized centers are circled by a yellow contour on the image, and the amount of γH2AX co-localization is reported for each image. Both exposure to γ-rays (Cs) and 1 GeV/amu Fe are illustrated for the two different time periods considered after exposure to radiation (1–4 min and 5–10 min). Averages for Cs are based on two independent experiments, whereas averages for Fe are based on four independent experiments. A *t*-test was performed, and a *p*-value of 0.01 was computed between the first and second time period considered for γH2AX (statistical significance noted on the graph as *).

We monitored the relative amount of co-localization of γH2AX or ATMp with 53BP1 (Figure 9). As previously shown for other cell types and markers [6,10,28–30], both markers show fast co-localization with 53BP1 RIF, independently of the radiation quality. Co-localization significantly increased from 44% to 64% for cells within the first 10 min following 1 Gy of 1 GeV/amu Fe. Representative images are shown in Figure 9B. It is important to note here that RIF frequencies following either high LET or low LET did not change appreciably during this time period (i.e., 2%–7%, all labels included). Therefore, it seems unlikely that a 20% increase of co-localization could be simply explained by more foci appearing in common regions of the nucleus.

### DNA Damage Does Not Appear To Elicit Chromatin Decondensation

**Figure 10:**
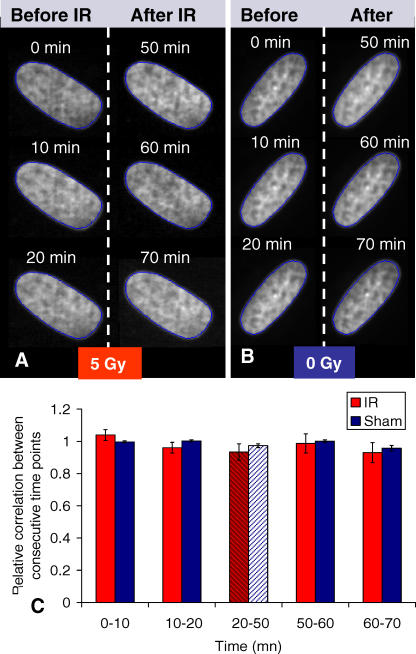
No Decondensation of Chromatin following 5 Gy of Low LET HeLa cells transfected with H1.2-GFP (see Materials and Methods) were imaged before and after exposure to 5 Gy of X-rays (A). Control cells that were not exposed to X-rays were also monitored (B). Intensity correlation within the nucleus (contour shown in blue on images) was computed between consecutive time points. To correct for noise which is unique to each image, correlation values were normalized to the average correlation measured for the three first time points (i.e., before radiation). These relative correlations are graphed in (C) for both groups (i.e., each group represents averages from five different nuclei). Correlation values before and after radiation (20–50 min) are shown on the graph by dashes on the bars for both control and irradiated specimen.

The above analysis suggests that either RIF occur at restricted locations or chromatin remodels as a result of DNA damage. Indeed, both 53BP1 and γH2AX are chromatin modifications. To test if the pattern of non-random distribution of RIF locations were due to global chromatin reorganization, we monitored chromatin in HeLa cells transfected with histone H1.2 fused to GFP. The chromatin pattern was monitored before and after 5 Gy of X-rays in the same cells. Representative time frames are shown in Figure 10. Chromatin patterns were unaffected by irradiation.

## Discussion

We previously developed computation models that simulate the production of DSB in hypothetical spatial geometries [22,23]. In this work, we extended such models to predict the pattern of radiation-induced DNA damage detected by protein markers in biological images after taking into account the optical properties of the microscopy. Two distinct radiation qualities were considered, low LET (i.e., photons) and high LET (i.e., 1 GeV/amu Fe ions) using this model. DSBs or clusters of DSBs appeared as foci in simulated images or pRIF). We found that the frequency pRIF matched the frequency of RIF from three different markers measured shortly (i.e., 4.5 min) after exposure to 1 GeV/amu Fe. Using these simulations, we noted that Fe tracks had many close-by DSBs that could not be resolved by optical microscopy, which led to larger and fewer foci along these pseudo tracks. This phenomenon was reported previously on experimental data for high-LET tracks [7,11]. The fact that frequencies were the same for pRIF and RIF in this case also suggests that when DNA damage extends over large regions in the nucleus it leads to a systematic and rapid formation of focal protein marks. Therefore, RIF are a good indication of DNA damage induced by HZE. In contrast, the frequencies measured for low-LET RIF from 4.5 to 60 min post-IR were reduced by more than 60% from what we predicted. RIF in this case is probably not a good DNA damage marker, suggesting that non-complex DSBs lead to a lower and/or slower RIF response.

We then introduced an image-based model that could predict DNA damages for low LET or high LET in real nuclear images. This was done by randomly reshuffling the locations of detected foci, using DAPI intensity of each pixel as the probability to have damage at that pixel location. After validating this approach on the simulated nuclei previously analyzed for pRIF frequencies, we applied it to real data. We observed that the majority of RIF along tracks were spaced by gaps larger than 1 μm, whereas our image-based approach predicted the majority of damages to be less than 1 μm apart. This was observed as early as 4.5 min post-IR and the deviation from prediction was even greater at 30 min following exposure. To determine whether this result reflected preferential locations of foci in the nucleus, we introduced a new set of imaging parameters that quantify the location of RIF relative to the nuclear DAPI pattern. Using this tool, we showed for both high-LET and low-LET radiation that RIF were more frequently located in low DNA density regions than predicted by image-based modeling. We also showed that RIF occurred predominantly at the interface between high and low DNA density regions. Interestingly, ATMp did not show as strong a trend as the other markers, supporting its early, but mobile, role in the DNA damage response [31]. Finally, we measured a rapid increase in the co-localization between different DNA damage markers over the first 10 min following exposure to both radiation qualities.

We conclude from these studies that nuclear organization plays an important role in the response to DNA damages. Specifically, foci locating preferentially in low DNA density regions suggest that damages occurring in condensed regions of the DNA are not always detected, leading to their lower proportion. Detection in condensed areas might then depend on either local decondensation of the chromatin as suggested by others [32,33] or movement of the damaged site to more open regions of the chromatin. We tend to be in favor of the DSB movement hypothesis for the following reasons. First, the rapid accumulation of RIF at the interface between high and low DNA density contradict what we know about the way radiation deposits its energy in tissue; second, imaging of HeLa cells transfected with histone H1.2 GFP exposed to a high radiation dose (5 Gy) of X-rays did not show a change in chromatin pattern compared with unexposed cells. Furthermore, it has been shown that some genes become transcriptionally active only upon relocating into open regions of the nucleus [34]. In fact, whole parts of a chromosome have been reported to be able to move over a 1–5 μm path within a few minutes during transcription activation in mammalian cells [35]. The only way to resolve these hypotheses unequivocally is to use live cell imaging of GFP-fused proteins recruited at the site of DNA damage such as NBS1 or 53BP1 exposed to physiological doses of ionizing radiation.

Another important aspect of DNA damage response to radiation provided in this work is the fact that damage appears to be spatially organized. Specifically, the lack of foci in close proximity along tracks suggests the existence of discrete self-excluding nuclear regions where DNA damages are clustered. This result supports the existence of “repairosomes” in mammalian cells, which has been suggested by Savage [36,37]. These nuclear domains would provide the necessary clamping and orientation for repair to take place but could also lead to translocations or chromosome aberrations when multiple breaks would be simultaneously processed. The existence of “repair centers” has already been shown in yeast Saccharomyces cerevisiae where Lisby et al. engineered a system of fluorescently marked DSB [38]. They showed in such a system that in 40% to 50% of the cases, two individual DSB relocalized into one common focus which was rich in Rad52 proteins. On the other hand, there is no direct evidence of repair centers in mammalian cells, although some reports have suggested their existence. For example, DiBiase et al. hypothesized that Ku proteins might recruit DSB to DNA-PKcs (catalytic subunit of DNA-PK) since it is fixed on the nuclear matrix, allowing fast DNA repair via nonhomologous end-joining [39]. More recently, it was also shown that MRN complex “tethers” damaged DNA to help activate ATM by increasing locally the concentration of DSBs [40]. Therefore, our work adds to this hypothesis by identifying for the first time in mammalian cells morphological features in the nucleus where protein markers of damage response preferentially locate. More work is needed to identify features that define these subnuclear regions.

## Materials and Methods

### Cell culture.

Human mammary epithelial cells (HMEC-184; 184v; passage 7–10) were cultured in serum-free medium as previously described [41]. HMEC-184 were irradiated with 1 Gy of ionizing radiation 2 d post-plating. Low-LET radiation exposures were conducted using a 5600 curie source of 137-Cs γ-radiation. The high-LET radiation was 1 GeV/amu Fe ions from the NASA Space Radiation Laboratory of Brookhaven National Laboratory. HeLa cells (ATCC) were cultured in DMEM with 10% FBS and exposed to a 160-kV X-ray source. In all three types of radiation, the same dose rate was used, i.e., 1 Gy/min.

### Reagents.

Primary: mouse monoclonal anti phospho-histone H2AX (Ser139) antibody (lot 27505, Upstate Cell Signaling Solutions, http://www.upstate.com/) used at 1.42 μg/ml; mouse monoclonal anti-phosphorylated (pS1981) ATM protein kinase antibody (lot 14354; Rockland, http://www.rockland-inc.com/) used at 2.15 μg/ml; rabbit polyclonal anti 53BP1 (lot A300-272A, Bethyl Lab, http://www.bethyl.com/) used at 5 μg/ml. Secondary antibodies were used at 1:300 (Dk anti-Rb Alexa 594, lot 40247A, and Gt anti-Ms Alexa 488, lot A11029, from Molecular Probes, Invitrogen, http://www.invitrogen.com). The H1.2 GFP construct was a generous gift from Dr. Michael Hendzel, University of Alberta, Canada [42].

### Immunofluorescence.

Cells were grown on tissue culture–treated LabTek eight-well chamber slides. Chambers were fixed at room temperature for 15 min using 2% paraformaldehyde followed by successive wash and permeabilization with 100% methanol for 20 min at 20 °C. Nonspecific sites were blocked using 1% BSA for 90 min. The cells were incubated 2 h at room temperature with primary antibodies in blocking buffer in a humidified chamber. Following washes, primary antibody binding was detected using species-appropriate fluorochrome-labeled secondary antibodies incubated for 1 h at room temperature. Nuclei were counterstained with DAPI (4′,6-Diamidino-2-Phenylindole) using 0.5 μg/ml. Slides were mounted in Vectashield (Vector Laboratories, http://www.vectorlabs.com/) and stored at −20 °C until evaluated.

### Image analysis.

Cells were viewed and imaged using a Zeiss Axiovert epifluorescence microscope (Carl Zeiss, http://www.zeiss.com/) equipped with a multiband pass filter and a differential wavelength filter wheel. Images were acquired using a Zeiss plan-apochromat 40× dry, with an NA of 0.95 and a scientific-grade 12-bit charged coupled device camera (ORCA AG Hamamatsu, 6.45 × 6.45 μm^2^ pixels). The image pixel size was measured to be 0.16 μm, but based on the NA of the objective, the actual resolution of the image in the FITC channel was ∼0.5 × 0.488/NA = 0.26 μm. All images were captured with the same exposure time so that intensities were within the 12-bit linear range. All image manipulation and analysis were done with Matlab (MathWorks, http://www.mathworks.com/) and DIPimage (image processing toolbox for Matlab, Delft University of Technology, The Netherlands).

For track analysis, Fe ion tracks were manually identified on the most-in-focus slice in a conventional image stack. Tracks were defined only if there were four or more foci within the nucleus, and if the cells on the stack slice had parallel tracks, reinforcing the assertion that the line in question was the result of a particle traversal and not a simple random alignment of points (see Figure 2). To keep track of the radial (perpendicular to the track) displacement of foci, a stripe of 0.8 μm width along each track was sampled for the maximum intensity in the direction perpendicular to the track. This led to a 1-D intensity curve with maximal intensities along the track. Herein this curve was called the “maximum intensity profile,” or simply the “1-D profile.” Using the intensity-inverted 1-D profile, foci were detected by searching for local minima along the track using watershed algorithms as shown in Figure 3. For cells exposed to γ-rays, 3-D images had to be acquired since foci were not restricted along a line anymore. This made maxima detection more difficult. To address this issue, we used a method similar to previous work [43] where a tophat morphological filter was used to enhance the intensity of spherical spots in the image. The resulting image was then intensity-inverted, and watershed algorithms were applied to detect minima as was done for the 1-D profile. In both 1-D and 3-D cases, the center of the RIF was determined as a pixel with the maximum intensity as sampled over the identified RIF. These central pixels were taken as RIF coordinates and utilized for *Rdna* and *Rgrad* computations (defined in the main text), and for co-localization analysis. More details on the methods of image processing and analysis are in Results.

### Generation of DSB by Monte Carlo technique in virtual nuclei.

The Monte Carlo algorithm utilized here is based on the probability of a DNA DSB at a given location in the nucleus being proportional to the DNA density and the dose (energy per unit mass) deposited at that location. As shown previously [20,24], the spatial DSB distribution is generated via a stochastic process given by

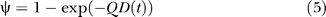
where *ψ* is a probability to create a DSB at a monomer (a small stretch of DNA containing 2 kbp of genomic information), *D*(*t*) is the local dose given by the track structure (it can vary sharply with the distance *t* from the track center), and *Q* is the constant determined from model fits to PFGE data. The correctly determined *Q* would generate proper DSB yields, fragment-size distribution functions, average numbers of DSB per nucleus per track, and the spatial distributions of DSB for several ions, any *E*, and any dose [20,22]. In this approach, the frequency of DSB depends on the properties of a track given by *D*(*t*) [18,44], but it will also depend on the DNA configuration given by a random walk model, as the probability *ψ* is applied to each monomer. In this model, a pixel can have a variable number of monomers corresponding to the density fluctuations of genetic material in the nucleus with high precision.

## Abbreviations

ATM: ataxia telangectasia mutated
ATMp: ATM phosphorylated at serine 1981
DAPI: nuclear dye 4′,6-diamidino-2-phenylindole
DSB: double strand break(s)
HZE: high energy particles
IR: ionizing radiation
LET: linear energy transfer (typical unit, keV/μm)
MRN complex: Mre11-Rad50-Nbs1 complex
PFGE: pulsed field gel electrophoresis
post-IR: following exposure to ionizing radiation
PSF: point-spread function
RIF: radiation-induced foci
γ-H2AX: histone H2AX phosphorylated at serine 139
